# Carrier Dynamics in Solution-Processed CuI as a P-Type Semiconductor: The Origin of Negative Photoconductivity

**DOI:** 10.1021/acs.jpclett.2c03720

**Published:** 2023-01-24

**Authors:** Robert Bericat-Vadell, Xianshao Zou, Mélio Drillet, Hugo Corvoysier, Vitor R. Silveira, Steven J. Konezny, Jacinto Sá

**Affiliations:** † Physical Chemistry Division, Department of Chemistry - Angstrom Laboratory, Uppsala University, Box 523, 751 20Uppsala, Sweden; ‡ Departments of Physics and Chemistry and Energy Sciences Institute, Yale University, 217 Prospect Street, P.O. Box 208120, New Haven, Connecticut06520-8120, United States; § Institute of Physical Chemistry, Polish Academy of Sciences, Marcina Kasprzaka 44/52, 01-224Warsaw, Poland

## Abstract

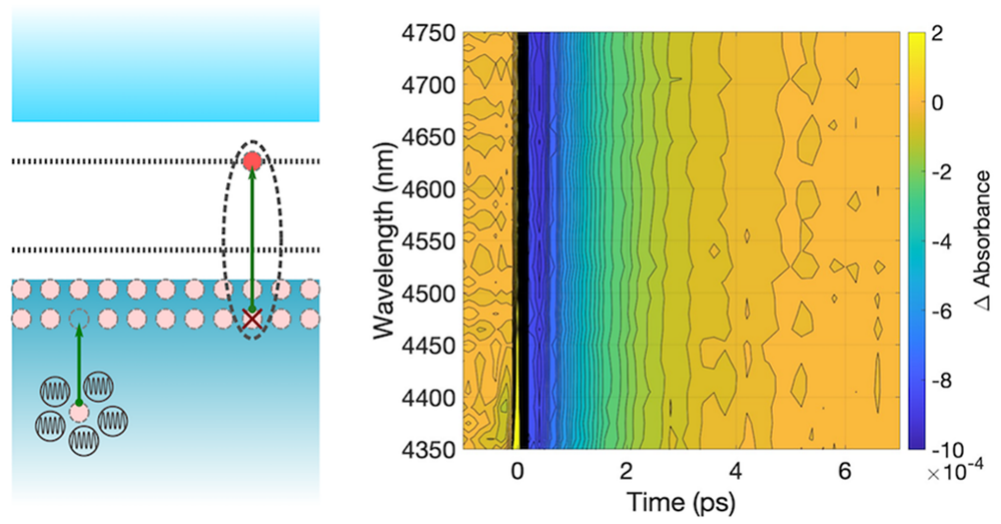

There is an urgent need for efficient solution-processable p-type semiconductors. Copper(I) iodide (CuI) has attracted attention as a potential candidate due to its good electrical properties and ease of preparation. However, its carrier dynamics still need to be better understood. Carrier dynamics after bandgap excitation yielded a convoluted signal of free carriers (positive signal) and a negative feature, which was also present when the material was excited with sub-bandgap excitation energies. This previously unseen feature was found to be dependent on measurement temperature and attributed to negative photoconductivity. The unexpected signal relates to the formation of polarons or strongly bound excitons. The possibility of coupling CuI to plasmonic sensitizers is also tested, yielding positive results. The outcomes mentioned above could have profound implications regarding the applicability of CuI in photocatalytic and photovoltaic systems and could also open a whole new range of possible applications.

Much research has been devoted to studying n-type semiconductors for photocatalytic and photovoltaic applications, while less attention has been paid to their p-type counterparts.^1−4^ This disparity has significantly affected the implementation of p-type semiconductors in photocatalytic and photovoltaic applications, despite their essential role. Copper (i) iodide (CuI) has been used n-i-p and p-i-n photovoltaic systems as hole transport layers,^5,6^ and in photocatalysts due to its better reductive capabilities compared to n-type ones.^3,7^ The bulk of the research on p-type semiconductor has been on nickel(II) oxide (NiO),^3,8^ which is a promising material but it has some disadvantages. For instance, NiO presents high recombination rates,^9^ relatively low carrier mobilities,^3^ absorption in the visible range due to Ni-oxide defects^3^ and potential health risks.^10^

CuI seems to avoid most NiO drawbacks, thus arising as an interesting alternative to NiO.^11^ Below 660 K, CuI is a p-type semiconductor with a zincblende structure and a direct 3.1 eV bandgap.^12,13^ CuI has high carrier mobilities and conductivity,^5^ high rates of interfacial electron transfer,^7^ chemical stability in various reaction conditions and solvents,^5,14^ abundant, nontoxic and environmentally friendly elements.^13,15^ Another significant advantage is that CuI can be processed from solution at environmental temperatures using simple and inexpensive equipment.^5,15^ High transparency in the visible spectral region can be obtained when prepared as a thin film.^6^

Herein, a detailed ultrafast dynamic of photoexcited free carriers study performed on solution-processed CuI is presented. The applicability of semiconductors to photovoltaics and photocatalysis is intimately related to the lifetime and mobility of the photogenerated carriers. Short carrier lifetime or high recombination rate is a determining reason for the low-efficiency devices.^16,17^ Recombination prevents the participation of the excited carriers in either a catalytic redox reaction or the generation of a photocurrent, wasting the energy obtained through light absorption.^18,19^ Likewise, carrier mobilities also play a crucial part. Mobility sets the average distance a carrier travels before recombining, i.e., the diffusion length.^20^ Longer diffusion lengths increase the chances that the carrier will reach a catalytic site or an electrode,^3,17^ consequently increasing the efficiency of the corresponding system.

This report aims to unveil the mechanisms behind the carrier dynamics after photoexcitation in CuI and also to determine the characteristic times of such processes, both through spectroscopic and photoconductivity measurements. As a whole, this study will contribute to a better understanding of the CuI properties and to a better assessment of its qualities as a p-type semiconductor for future potential applications.

The CuI thin film was prepared as described elsewhere.^11,15,21^ Briefly, approximately 150 μL of a saturated solution of CuI in acetonitrile (35 mg/mL)^22^ was spin-coated on an ozone cleaned 20 mm × 20 mm cover glass at 2000 rpm for 30 s. After letting the film dry for 1 min, a new layer is spin-coated on top with the same parameters. The films were composed of 6 to 10 spin coat cycles depending on the measurements. The CuI thin films were annealed at 373 K for 10 min after all the coatings.

The UV–vis spectrum (Figure 1) shows the characteristic absorption edge of the CuI. Annealing led to slight decrease in the bandgap energy from 2.90 to 2.88 eV. It is also clear that the annealing process improves the optical properties of CuI as this enables the detection of the excitonic peak Z_1,2_ (at 410 nm or 3.02 eV).^12^ This change is in line with previous observations,^13^ which were rationalized as an indication of improved film crystallinity since broader and ill-defined excitonic peaks are characteristic of poorer crystallinity^23^ or small crystallite sizes.^24,25^ Although annealing appears to improve the bulk crystallinity, it is to be expected that the heating of CuI above 333 K induces vacancy formation.^15^ Iodine vacancies (V_I_) act as electron donors, compensating the native acceptor impurities in CuI, namely, copper vacancies (V_Cu_). The compensation decreases the hole concentration,^15^ decreasing the material’s conductivity. In addition, V_I_ can behave as trap states and hole scatters, reducing the corresponding carrier mobility.^15^ The Fourier-transformed infrared (FTIR) spectrum of the annealed CuI film (Figure S1) shows a monotonic and featureless absorption that increases in intensity with increased wavelength. Such a spectral feature is characteristic of free carriers in the case of holes generated by the ionization of V_Cu_ at room temperature.

**Figure 1:**
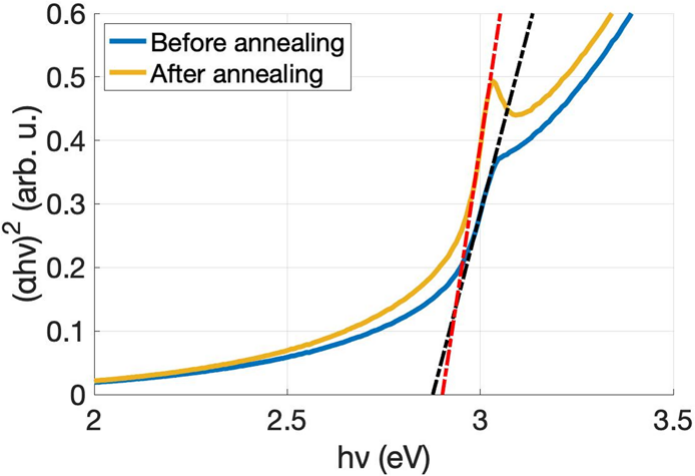
Optical absorption of CuI film (10 layers) before and after annealing at 373 K.

Carrier dynamics in semiconductors involve processes, such as photoexcitation, relaxation, trapping, etc., that take place on subnanosecond time scales.^20^ These processes can be studied directly through ultrafast methods such as time-resolved infrared absorption spectroscopy (TIRAS in pump–probe mode) since free carriers display a broad featureless signature in the mid-infrared range.^26^

The free carrier decay, the kinetics of the free carriers’ trapping and recombination processes, was monitored after interband photoexcitation at 400 nm and probed in the 4350–4750 nm range. The resulting transient color map is shown in Figure 2a. A kinetic trace was extracted at 4550 nm to illustrate the carriers’ behavior (Figure 2b). As can be observed in Figure 2a and more clearly in Figure 2b, after time zero, there is a pronounced positive absorbance difference (Δ*A*) that drops sharply to negative values reaching its maximum amplitude at 500 fs, after which the signal recovers to positive Δ*A* within 4 ps. The signal follows an exponential decay afterward. Note that the shape of the kinetic trace cut is nearly identical to all the probing wavelengths, as expected for a free carrier signal.

**Figure 2:**
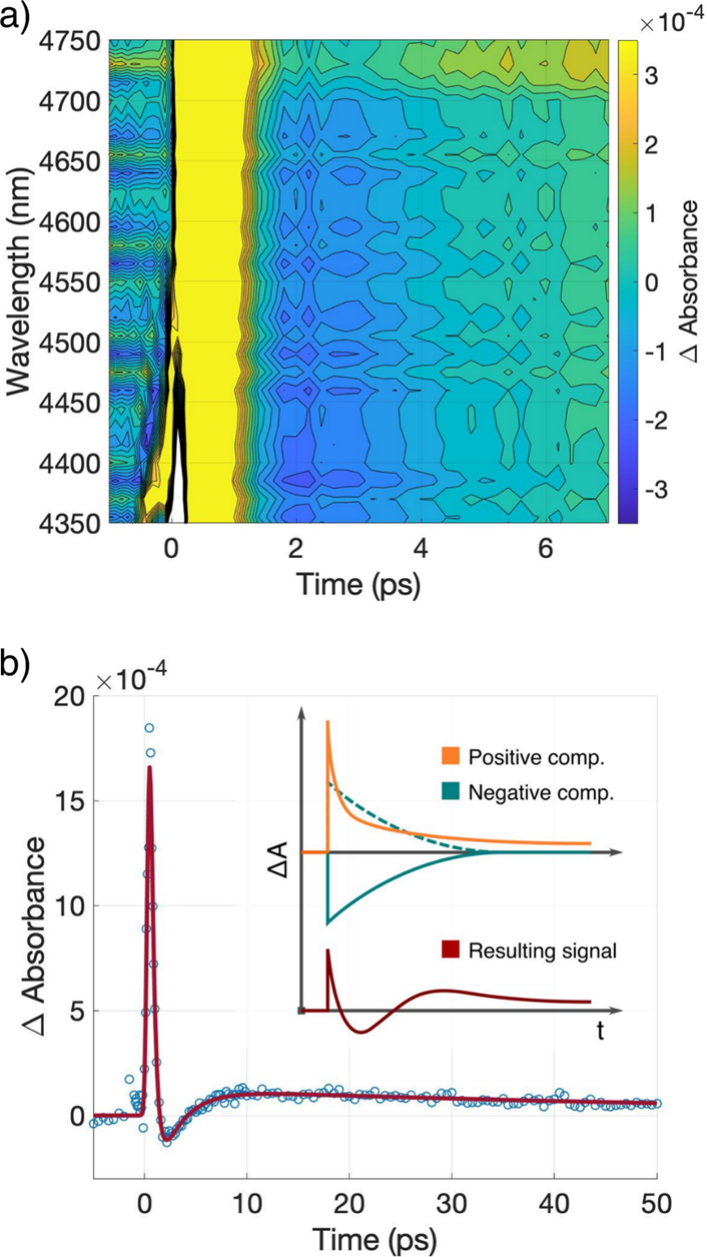
CuI TIRAS measurement after bandgap excitation at 400 nm. (a) Contour plot showing a broad featureless signal after 0 ps indicative of free carriers and (b) kinetic trace extracted at 4550 nm. The inset depicts a schematic representation of the deconvolution of the two components that combined generate the observed kinetic trace shape. The dashed trace is the modulus of the negative component to help visualize the detected signal. The laser fluence was 17.6 μJ/cm^2^.

The featureless response in the mid-IR Δ*A* is characteristic of changes in the free carrier densities or mobilities after photoexcitation.^27^ In more detail, the classical expression for free-carrier absorption is^28,29^
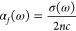
1where, *α*_*f*_(ω) is the free carrier absorption coefficient, *n* is the refractive index, *c* is the speed of light and σ(ω) is the AC conductivity. In the Drude formalism, by considering that for a probe in the 4000–5000 nm range ω^2^τ_*c*_^2^ ≫ 1, (ω is the angular frequency and *τ*_*c*_ the carrier mean free path), σ(ω) can be expressed as^29^
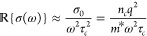
2where σ_0_ corresponds to the DC conductivity, *q* corresponds to the carrier charge, *n*_*c*_ is the carrier population density, and m* is the carrier effective mass. From eq 1 and eq 2, a last relation can be established:
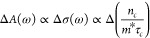
3Thus, photoinduced increments in *n*_*c*_ will translate in a positive Δ*A* (positive photoconductivity response (PPC)), while increments in m* and *τ*_*c*_ will yield negative Δ*A* (negative photoconductivity response (NPC)).^30,31^

The kinetic trace extracted at 4550 nm was successfully fitted with three exponential decays, two of them with a positive amplitude (τ_1_ ≈ 0.4 ps (98%) and τ_2_ ≈ 60 ps (2%)) and one with a negative amplitude (τ_3_≈ 2 ps). A schematic representation of the signal deconvolution is presented in the Figure 2b inset. This positive trace corresponds to the free carriers originating from photoinduced interband transitions. The decays relate to charge recombination, with a short lifetime associated with charge recombination shortly after charge separation and the longer lifetime to charge recombination that managed to escape the initial process. The negative component can be justified in terms of a decrease in *n*_*c*_, or an increase in m* and *τ*_*c*_. No matter the origin, negative Δ*A* are rare in 3D semiconductors. Generally, photoexcitation leads to the generation of free carriers in great numbers,^30^ outweighing any influence that a change in m* or *τ*_*c*_ could have, i.e., PPC signal.^27,32^ NPC responses are more common in metals and semimetals commonly detected in the THz range.^27,30^

An experiment exciting at 550 nm, well below the threshold for interband transitions, was used to separate the negative component from the positive ones (Figure 3a,b). This excitation wavelength suppresses free carrier formation thus permitting the study of the isolated NPC signal, which is evident on the kinetic trace presented in Figure 3b. The insert in Figure 3b shows the NiO behavior in the same experimental conditions for comparison. In the case of NiO, there is a positive signal when exciting below the bandgap energy indicating free carrier formation due to excitation of trap states. The CuI signal shows solely a negative contribution, i.e., no free carrier formation. The kinetic trace was fitted with one exponential decay with lifetimes of τ_1_≈ 1 ps, similarly to the NPC when exciting across the bandgap.

**Figure 3:**
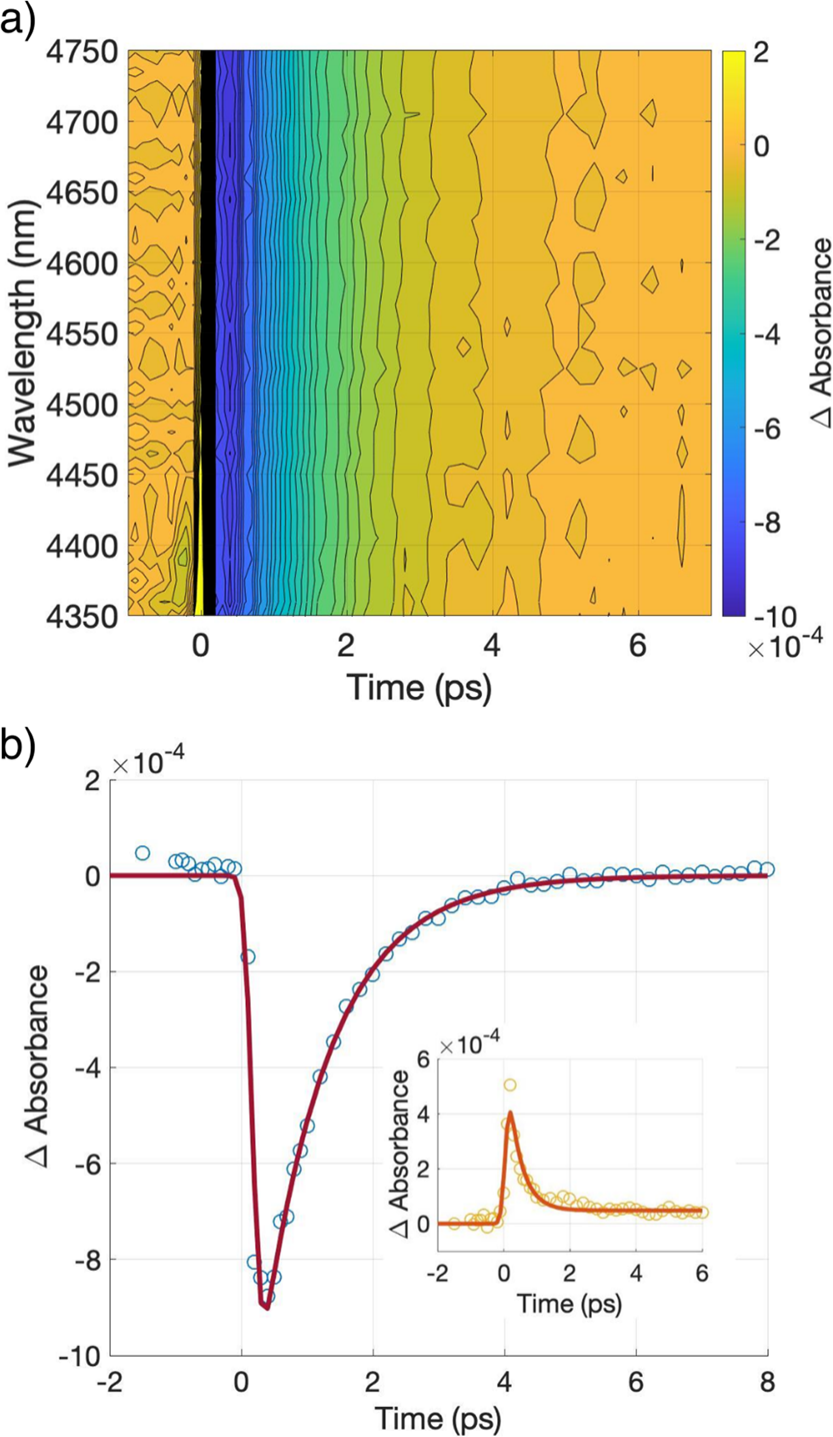
CuI TIRAS measurement after bandgap excitation at 550 nm. (a) Contour plot showing a broad featureless negative signal after time zero and (b) kinetic trace extracted at 4550 nm. The inset depicts the signal related to NiO measured in the same conditions for comparison. The laser fluence was 13.5 μJ/cm^2^.

To help the understanding of the signal, complementary conductivity measurements with and without light were performed. Figure 4 shows a decrease in activation energy (*E*_a_) with light estimated through the Arrhenius plot, and found to be 25.9 and 16.0 meV for the sample under dark and white light illuminated, respectively. Figure 4 also depicts a drop-in conductivity at room temperature when the sample is under illumination. This last observation is in line with the NPC response, corroborating the origin of the TIRAS signal. Therefore, several models will be devised in the following paragraphs to explain the unusual ultrafast NPC response in CuI. Figure 5 shows a schematic representation of the proposed models.

**Figure 4:**
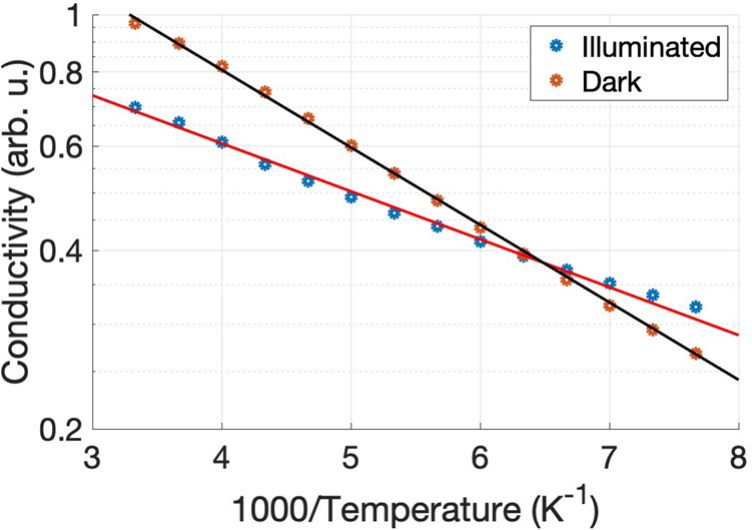
Conductivity of CuI at different temperatures in the dark and under white light illumination. The data are plotted as an Arrhenius plot used to estimate the activation energy (*E*_a_).

**Figure 5:**
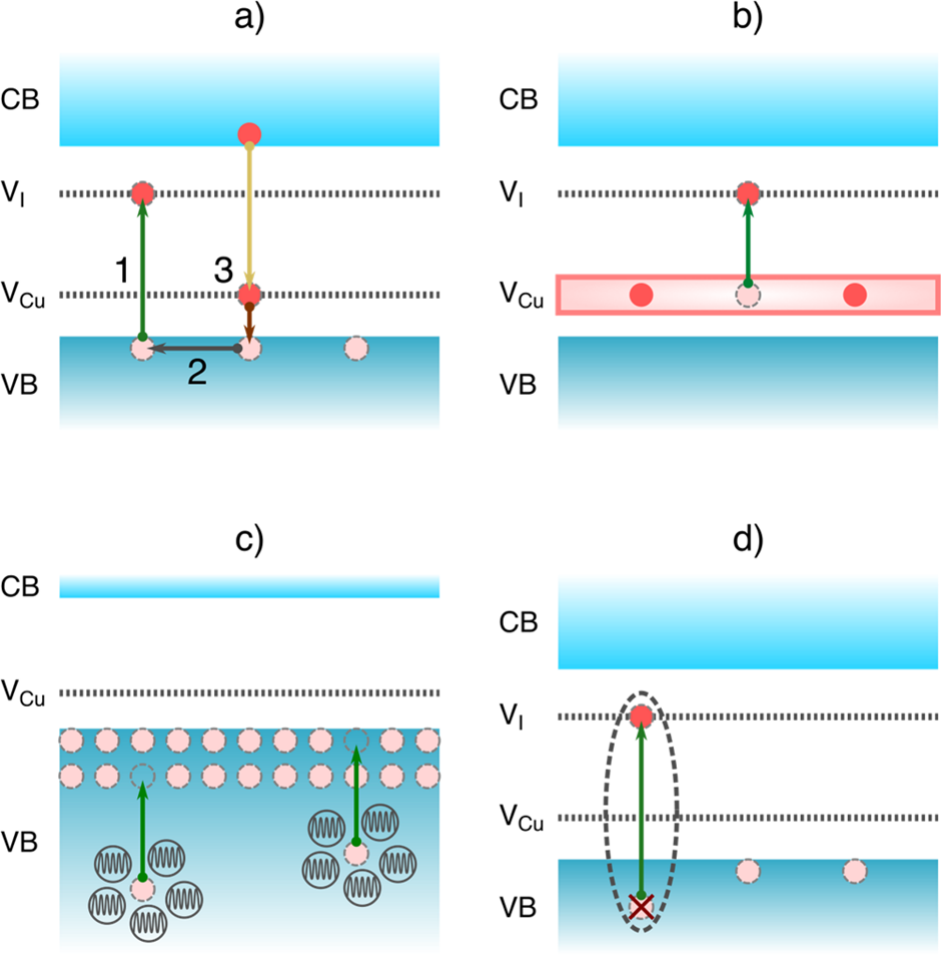
Possible mechanisms behind the negative TIRAS Δ*A* on CuI. (a) Combination of transitions between the bands and trap states leading to a transient decrease in the number of free carriers; (b) transitions from a metallic impurity band to the trap states below the CB also leading to a decrease in number of free carriers; (c) intraband transitions of the free carriers (holes) promote the formation of polarons, which in turn increase the hole’s effective mass, and (d) CuI excitation leading to the formation of strongly bounded excitons, which has the effect of reducing the number of electrons in the VB and, therefore, decreases the effective hole absorption.

The first model involves a series of transitions between the valence band (VB), conduction band (CB), and trap states of CuI (Figure 5a). These transitions would transiently decrease the population of free carriers, and consequently yield an NPC response. Briefly, an electron in the VB is photoexcited into an ionized donor level (1); the hole left at the VB diffuses until it is promoted to an ionized acceptor (2); there, the photoexcited hole and a free electron originating from an ionized donor recombine (3), effectively decreasing the number of free carriers in the process. Here, two stages can be differentiated: during the photoexcitation new free carriers are generated (holes in the VB), increasing their total number; only after recombination (step 3) is there a decrease with regard to the unexcited case. Therefore, the resulting signal should present the first rise in Δ*A* followed by a drop to negative values,^33^ which is not consistent with what was observed in Figure 3, where only a negative component is present. One might argue that the time resolution (ca. 120 fs) is not sufficient to detect the initial positive rise. Still, according to published literature, the expected time scales for the rise are in the range of microseconds to seconds,^33,34^ far from the picoseconds response observed herein. Finally, according to the values provided in literature for the V_Cu_ and V_I_ energies,^35^ the acceptor levels in CuI lay closer energetically to the VB than the donor levels are to the CB. This implies that the excitation in (1) is less energetic than the electronic decay in (3), i.e., if this model holds, the material would be emitting more energy than it would be absorbing, something impossible in terms of energy conservation law.

The second model postulates the existence of an impurity band separated from the CB and the VB (Figure 5b). Impurity bands appear in materials with a high concentration of impurities, dopants, or defects. After a certain concentration threshold, named the insulator to metal transition, the wave functions of these impurities start to overlap with each other, and their associated carriers become delocalized.^36^ If the defects and impurities energy states are placed energetically far from the CB, and the VB, a metallic band made of impurity states can arise inside the bandgap.^36^ It has been suggested that carriers in the impurity band should have similar spectral features as the free carriers in the VB and CB.^37−39^ If carriers inside the impurity band are considered analogous to free carriers, a simple mechanism can be devised to justify the measured negative component: when carriers in the impurity band are excited into localized states, the free carrier density decreases and, therefore, also does the free carrier absorption. This model could also explain literature data showing a decrease in the width of the acceptor band (V_Cu_) in CuI with increasing heat-treatment temperatures.^40^ In this case, the exposure of CuI to higher temperatures should minimize the spectral features related to the presence of an impurity band, which is consistent with the behavior observed in Figure 6. However, this model raises the main concern: to allow for free carrier absorption (i.e., intraband transitions) in the 4000–5000 nm range, the impurity band should be at least as broad as 0.31 eV. Such energy distribution appears to be exceptionally wide for an impurity band, considering that previous literature estimated widths around 0.07 eV for acceptor bands in CuI.^40^

**Figure 6:**
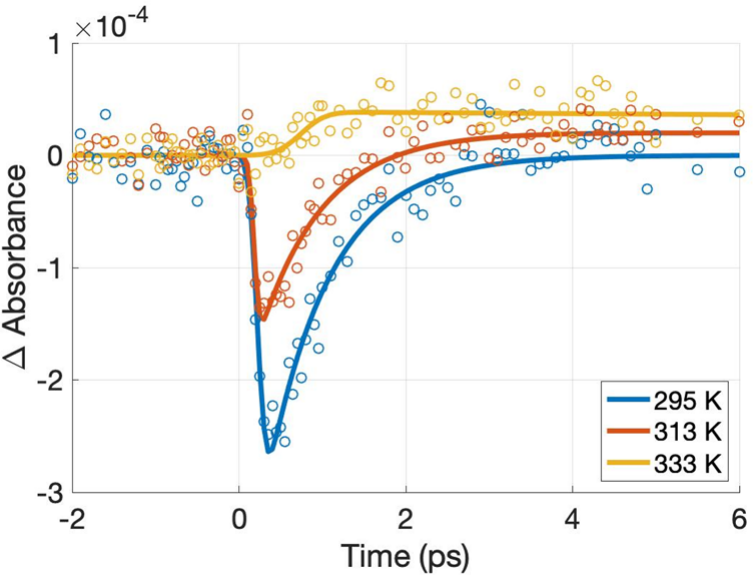
Temperature dependence of the kinetic trace extracted at 4550 nm after 550 nm excitation. The laser fluence was 14.6 μJ/cm^2^.

The third model considers polaron formation (Figure 5c). After excitation, hot carriers can be formed through intraband transitions, which subsequently relax, generating phonons.^27,32^ In some materials, the phonons generated in this way can strongly couple with the decaying hot carriers.^41,42^ The new particles arising from this short-range coupling are polarons and can present a substantially higher m* than the free carriers.^41^ As seen from eq 3 an increased m* induces a NPC response.

Different materials’ excitation can lead to different types of polarons; two important classifications in the present context are distinguished as large polarons and small polarons.^43^ Large polarons have the secondary effect of “protecting” the carrier against scattering,^44^ effectively increasing *τ*_*c*_ and strengthening NPC signal. Nevertheless, the formation of small polarons could also account for the NPC signal since small polaron mobilities are reduced with decreasing temperatures. This implies that, in materials where small polarons are present, the NPC response becomes more prominent at lower temperatures. For either of the two types of polarons in this model, after some time, the material starts cooling down, the electrons and phonons conforming the polarons start to dissociate,^41^ and the excess phonons dissipate into the environment,^32^ recovering that way the original conductivity Δ*A* = 0).

There is a key argument against this model: the probability of free carrier absorption is low for an excitation wavelength of 550 nm, making the generation of hot carriers highly inefficient. In addition, this a priori weak mechanism would compete with the simultaneous generation of the free carrier due to transitions involving trap states, which could generate a relevant PPC signal. It should be mentioned why variations in *τ*_*c*_ alone cannot account for the NPC response, as proposed for the negative THz absorption in metals^27^ and semimetals.^32,45^ For these materials, photoexcitation also increases the number of free carriers. However, in relative terms, the change is not appreciable, as the intrinsic carrier density of the material is already high. Thus, variations in carrier mobility are much more relevant.^32^ As mentioned previously, one byproduct of photoexcitation is the generation of phonons. These phonons can act as scatters, decreasing the *τ*_*c*_ of the free carriers.^27^ In the far THz regime, where ω^2^τ_*c*_^2^ ≪ 1, the relation Δ*A*(ω) ∝ Δ*τ*_*c*_ holds and, therefore, decreases in *τ*_*c*_ because of an increased scattering rate with phonons lead to NPC responses. This is not true for wavelengths in the 4000–5000 nm range, where Δ*A*(ω) ∝ Δτ_*c*_^–1^ and, therefore, a decrease in *τ*_*c*_ leads to PPC responses. Considering that, unless coupled to another mechanism, rises in *τ*_*c*_ are unlikely in the context of a photoexcitation, this alternative seems unlikely.

Jin et al.^46^ observed large polaron formation on CH_3_NH_3_PbI_3_ (MAPbI_3_) perovskite in large grains and thin films. The large polarons were formed within 500 fs in the grains and protected the charge carriers from being trapped, effectively increasing their lifetime. However, in their solution-processed MAPbI_3_ thin films, the polaronic effect was less significant, with the photoconductivity showing a faster decay, suggesting a higher degree of charge trapping in defects and imperfections. Their lifetime in the thin films is within the range detected here, supporting the idea of large polaron formation. Moreover, the polarons reduced mobility compared to free carriers supports our proposal for the origin of NPC. Similar observations were reported from thin films of CsPbBr_3_ perovskites by Cinquanta and co-workers.^47^

The fourth model involves the formation of strongly bound excitons (Figure 5d). In a material where the Coulombic interaction between the hole and electron pair is strong, such as in the case of CuI, photoexcitation could give rise to bound excitons (CuI exciton binding energy is ≈62 meV).^48^ Li et al.^49^ observed an ultrafast negative transient absorption signal at 3.7 eV (0.65 eV higher energy than the bandgap bleach signal), which they assigned to free excitons, confirming the existence of strongly bounded excitons. Excitons are quasi-particles formed by bound hole–electron pairs, which in the case of CuI excited at 550 nm, would appear when promoting an electron from the VB to a trap state. In this case, the hole left in the VB would not be regarded as a free carrier, as it would still be bound to the localized electron. However, the creation of the exciton removes one electron from the valence band while the number of free holes remains constant. This reduction in the number of electrons in the VB can affect the effective absorption of the free holes since, after excitation and exciton formation, fewer electrons are available to be promoted into the free state represented by the hole. Usually, the effect of decreasing the population density of electrons in the VB or of the free states in the CB is not considered, mainly due to the large unbalance between their population and the population of electrons in the CB and holes in the VB. Despite this, for some cases where the VB in the semiconductor is already highly depopulated, the depletion of electrons from the valence band could be the reason behind an NPC response.

As CuI is often used as a hole-transporting material, not as a photoactive material, it is essential to establish if the unique NCP response is also present when CuI is coupled with a light absorber, such as gold plasmonic nanoparticles (Au NPs).^26,50,51^ For these experiments a new CuI thin film was prepared, where Au-NPs (5–6 nm as determined by dynamic light scattering and electron microscopy) were sprayed in between every CuI spin coat cycle. As seen in the UV–vis spectrum of an Au–CuI film in Figure S2, there is a broad absorption peak centered approximately at 550 nm assigned to the Au NPs. The carrier dynamics were measured again in the 4350–4750 nm range using a 550 nm excitation pump. The resulting dynamics at 4550 nm are shown in Figure 7.

**Figure 7:**
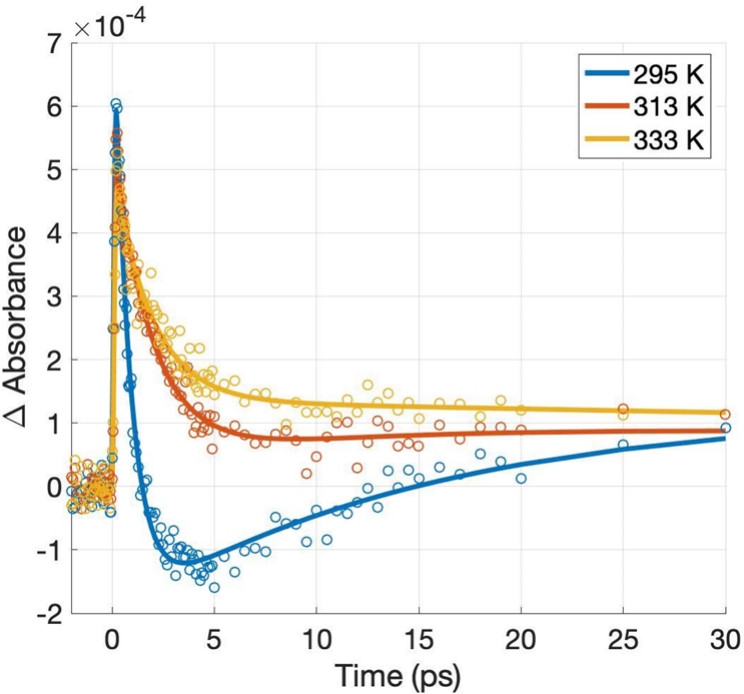
Temperature dependence of the kinetic traces extracted at 4550 nm after 550 nm excitation of Au–CuI film. The laser fluence was 14.6 μJ/cm^2^.

At room temperature (295 K), a similar kinetic feature was observed when performing interband excitation, which was absent when doing intraband excitation (at 550 nm). The positive component relates to hole injection from the Au-NPs into CuI, confirming the successful sensitization of the CuI by Au. The positive signal is overlapped with the NPC signal prevenient from CuI, as discussed previously, confirming its participation in the photosystem dynamics, even in a sensitized film. At higher temperatures, the amplitude of the negative component decreases in absolute value, in agreement with what was observed in Figure 6, confirming the suppression of the NPC signal at higher temperatures also in the sensitized film. It is also worth noting that the amplitude of the positive component (hole injection) also decreases with temperature, indicating that the efficiency of the hole injection process decreases with an increase in temperature. This result contrasts with what was observed in plasmonic hot electron injection efficiencies, which were promoted with increased temperature.^52^ This behavior confirms the theoretical prediction of hot carrier generation in plasmonic nanoparticles, which suggests that the energetic difference between the hot electron and hole generated after the plasmonic dephasing is predicted to be the same as the energy of the exciting radiation.^53^ If this difference is expected to remain constant, increasing the hot electron energy with increasing temperatures implies a concomitant decrease in the hot hole energy.

In conclusion, the free carrier dynamics in solution-processed CuI have been explored through TIRAS and conductivity measurements. Specifically, the response of CuI to photoexcitation was studied at energies above and below the threshold for interband transitions. The signal is a convolution of ultrafast positive and negative Δ*A* IR contributions (PPC and NPC responses, respectively) for the energies above the threshold. Although the first response is typical in semiconductors, the second is far more exotic. For energies well below the threshold for interband transitions, only the negative component remained, and it was shown to be temperature dependent. Different models are presented to justify such an unexpected signal, with the polaron formation or formation of strongly bound excitons being the ones that better explain the experimental observations. In general, semiconductors displaying NPC responses have attracted significant interest due to their potential applications, specifically in optoelectronics^30^ and sensing devices.^54^ However, the behavior was also found on sensitized CuI films that have implications for photocatalysis and photovoltaic applications. The drop in free carrier densities or mobilities behind the NPC response could reduce the chances for a photoexcited carrier to reach either a catalytic site or an electrode, reducing the overall device efficiencies.

## Supporting Information Available

A brief description of the experimental setups and conditions used to measure TIRAS and conductivity (PDF)
Transparent Peer Review report available (PDF)
