# Supplementary material for: Carrier Dynamics in Solution-Processed CuI as a P-Type Semiconductor: The Origin of Negative Photoconductivity
Source: J Phys Chem Lett. 2023 Jan 24;14(4):1007–13. doi: 10.1021/acs.jpclett.2c03720 (PMC9900634; doi:10.1021/acs.jpclett.2c03720)
Supplement: Supplementary file 1 — jz2c03720_si_001.pdf [file jz2c03720_si_001.pdf]

Supporting information

Carrier Dynamics in Solution-processed CuI as a P-type Semiconductor: the Origin of Negative Photocondutivity

Robert Bericat-Vadell,[a] Xianshao Zou,[a] Mélio Drillet,[a] Hugo Corvoysier,[a] Vitor R. Silveira,[a] Steven J. Konezny[b], and Jacinto Sá[a,c]*

[a] Physical Chemistry Division, Department of Chemistry - Angstrom Laboratory, Uppsala University, Box 523, 751 20 Uppsala, Sweden.

[b] Departments of Physics and Chemistry and Energy Sciences Institute, Yale University, 217 Prospect Street, P.O. Box 208120, New Haven, Connecticut, 06520-8120, USA.

[c] Institute of Physical Chemistry, Polish Academy of Sciences, Marcina Kasprzaka 44/52, 01-224 Warsaw, Poland.

* Email: jacinto.sa@kemi.uu.se

Transient infrared absorption spectroscopy (TIRAS)

Briefly, a 40 fs pulsed laser with a 3 kHz repetition rate was generated through the Libra Ultrafast Amplifier System designed by Coherent. Two optical parametric oscillators (TOPAS- prime, Light Conversion) are used to generate either the excitation beam and/or the probe light in the Mid-IR (3000-10000 nm). The signals were detected with an Horiba iHR 320 spectrometer. The pump laser power was constantly monitored with less than a 2% standard deviation. The timing resolution, i.e., the instrument response function is ca. 120 fs.

Meaningful fluence-dependent measurements were not feasible because the films were unstable. There was clear damage signs at higher fluencies that could not be avoided by

moving the laser spot. Moreover, the films were not homogeneous, restricting where one could perform the measurements in the film. This is expected due to the fast crystalization of the CuI film, as observed by several reports, including Wang e*t al. J. Mater. Chem. A* **2018**, *6*, 21435.

Conductivity measurements

Conductivity measurements were performed on sapphire/CuI/Au devices inside a Janis closed-cycle He cryostat using an Agilent B1500A semiconductor analyser.

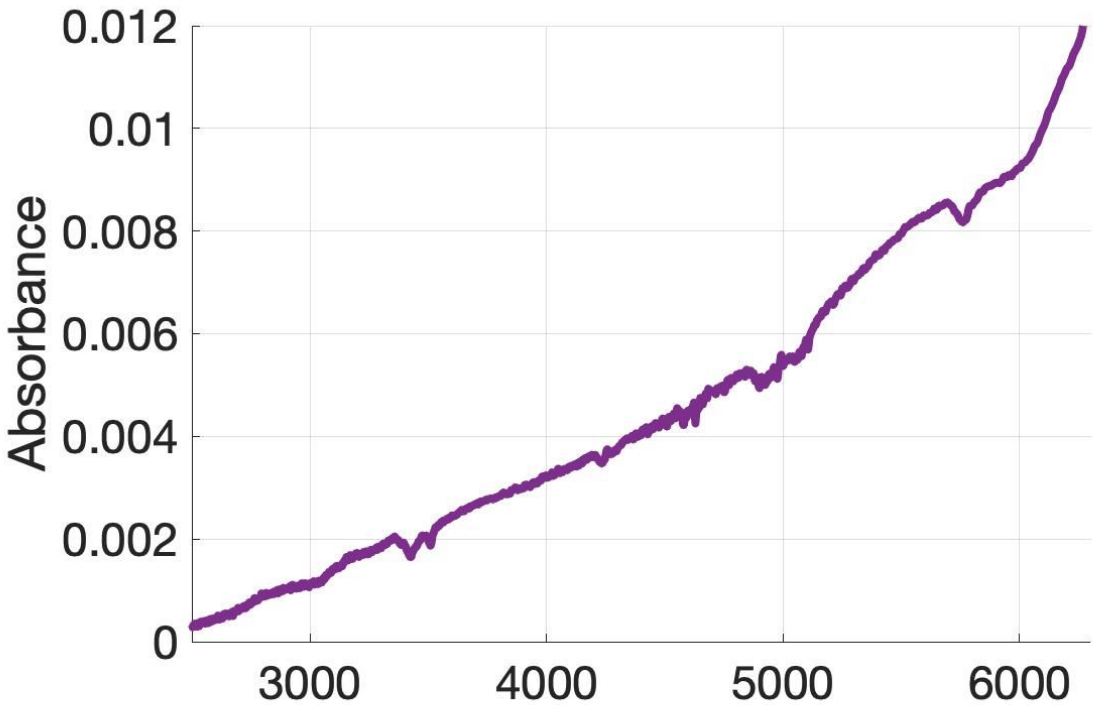

Figure S1. Fourier-transformed infrared (FTIR) spectrum of CuI film annealed at 373 K

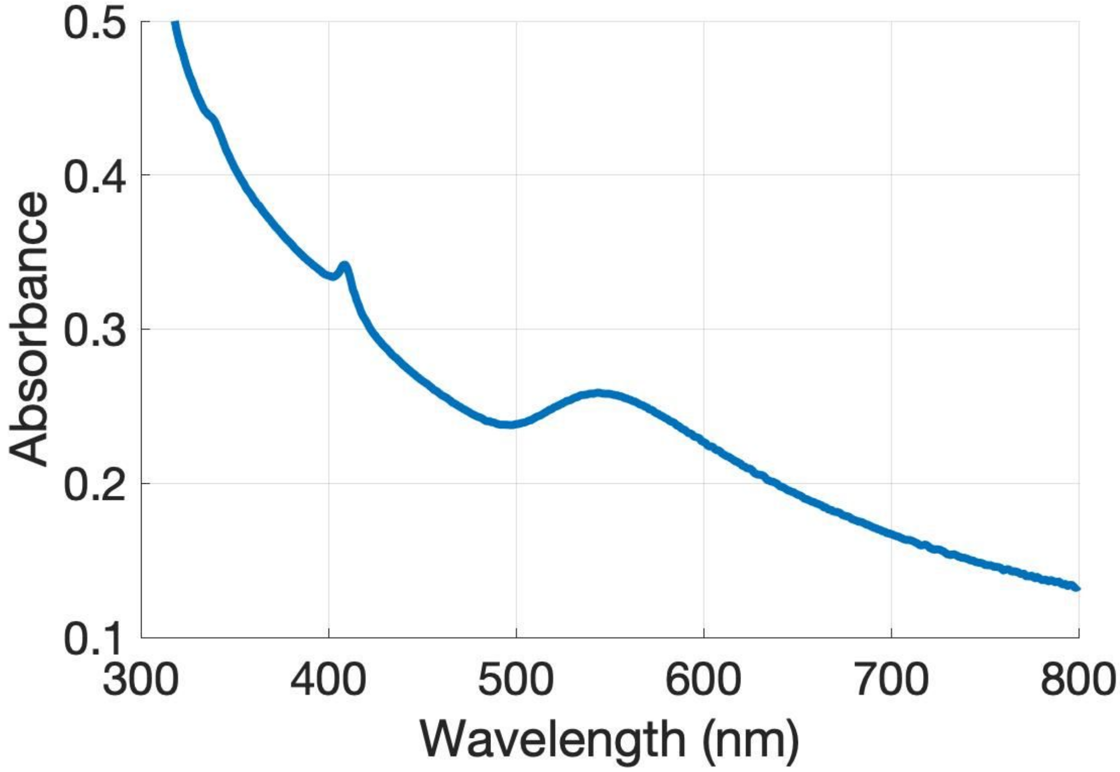

Figure S2. UV-Vis absorption of Au/CuI film.
