# Supplementary material for: Carrier Dynamics in Solution-Processed CuI as a P-Type Semiconductor: The Origin of Negative Photoconductivity
Source: J Phys Chem Lett. 2023 Jan 24;14(4):1007–13. doi: 10.1021/acs.jpclett.2c03720 (PMC9900634; doi:10.1021/acs.jpclett.2c03720)
Supplement: Supplementary file 2 — jz2c03720_si_002.pdf [file jz2c03720_si_002.pdf]

jz-2022-037206.R1

Name: Peer Review Information for "Carrier Dynamics in Solution-Processed CuI as a p-Type Semiconductor: the Origin of Negative Photoconductivity"

First Round of Reviewer Comments

Reviewer: 1

Comments to the Author

CuI is a promising p-type wide gap semiconductor. The authors provided a comprehensive study on the carrier dynamics of solution-processed CuI thin films. Overall, this manuscript has been well prepared, and I believe it will be very helpful to researchers who are investigating CuI materials and devices. However, I noticed that the ultrafast carrier dynamics of copper iodide thin films has been reported this year (Nature Communications 13, 6346, 2022), so I suggest the authors to compare their findings with this report.

Reviewer: 2

Comments to the Author

Robert Bericat-Vadell et al reported carrier dynamics in solution-processed CuI as a p-type semiconductor. Carrier dynamics after bandgap excitation yielded a convoluted signal of free carriers (positive signal) and a negative feature, which was also present when the material was excited with sub-bandgap excitation energies. This previously unseen feature was found to be dependent on measurement temperature and attributed to negative photoconductivity. The polaron formation and/or formation of strongly bound excitons being the ones that better explain the experimental observations. I recommend the paper be published in The Journal of Physical Chemistry Letters once the authors have answered the following questions/issues:

(1) Although the bibliography of the paper is already consequent, some key previous references dealing with the large polaron are missing. See e.g. Jin et al. Light: Science & Applications (2022) 11:209, and Cinquanta, E. et al. Phys. Rev. Lett. 122, 166601 (2019). These references should be quickly discussed and add in the revised version of the paper.

(2) The authors should rapidly discuss the dashed curve in the inset of Figure 2.

(3) I suggest that the authors should give more pump fluence dependent measurements and discussion in the paper.

(4) Please provide more information and discussion on the different temperature dependence of the positive and negative components in Fig. 6 and Fig.7.

Author's Response to Peer Review Comments:

Answers to Reviewers' Comments:

Reviewer: 1

CuI is a promising p-type wide gap semiconductor. The authors provided a comprehensive study on the carrier dynamics of solution-processed CuI thin films. Overall, this manuscript has been well prepared, and I believe it will be very helpful to researchers who are investigating CuI materials and devices.

We thank the Reviewer for the recognition of the submitted work's worthiness and novelty,as well as the endorsement for publication. We would also like to thank the Reviewer's time dedicated to the revision and helpful comments. We have addressed them below and updated the manuscript in accordance.

However, I noticed that the ultrafast carrier dynamics of copper iodide thin films has been reported this year (Nature Communications 13, 6346, 2022), so I suggest the authors to compare their findings with this report.

We thank the Reviewer for this comment. We looked into the suggested paper and updated the manuscript text and reference list accordingly. The paper clearly shows the presence of strongly bounded excitons that support one of our models.

We added to the text: "*Li et al. observed an ultrafast negative transient absorption signal at 3.7 eV (0.65 eV higher energy than the bandgap bleach signal), which they assigned to free excitons, confirming the existence of strongly bounded excitons.*"

Reviewer: 2

Robert Bericat-Vadell et al reported carrier dynamics in solution-processed CuI as a p-type semiconductor. Carrier dynamics after bandgap excitation yielded a convoluted signal of free carriers (positive signal) and a negative feature, which was also present when the material was excited with sub-bandgap excitation energies. This previously unseen feature was found to be dependent on measurement temperature and attributed to negative photoconductivity. The polaron formation and/or formation of strongly bound excitons being the ones that better explain the experimental observations. I recommend the paper be published in The Journal of Physical Chemistry Letters once the authors have answered the following questions/issues.

We thank the Reviewer for the recognition of the submitted work's worthiness. We have updated the manuscript with the Reviewer's suggestions. We hope the revised version removes any concerns the Reviewer might have had, and we can get the final endorsement for publication. We would also like to thank the Reviewer's time dedicated to the revision and helpful comments.

(1) Although the bibliography of the paper is already consequent, some key previous

references dealing with the large polaron are missing. See e.g. Jin et al. Light: Science & Applications (2022) 11:209, and Cinquanta, E. et al. Phys. Rev. Lett. 122, 166601 (2019). These references should be quickly discussed and add in the revised version of the paper.

We thank the Reviewer for this comment. We looked into the suggested papers and updated the manuscript text and reference list accordingly.

We added to the text: "*Jin et al. observed large polaron formation on $CH_3NH_3PbI_3$ ($MAPbI_3$) perovskite in large grains and thin films. The large polarons were formed within 500 fs in the grains and protected the charge carriers from being trapped, effectively increasing their lifetime. However, in their solution-processed $MAPbI_3$ thin films, the polaronic effect was less significant, with the photo-conductivity showing a faster decay, suggesting a higher degree of charge trapping in defects and imperfections. Their lifetime in the thin films is within the range detected here, supporting the idea of large polaron formation. Moreover, the polarons reduced mobility compared to free carriers supports our proposal for the origin of NPC. Similar observations were reported from thin films of $CsPbBr_3$ perovskites by Cinquanta and co-workers*."

(2) The authors should rapidly discuss the dashed curve in the inset of Figure 2.

We thank the Reviewer for this comment.

We added the figure caption: "*The inset depicts a schematic representation of the deconvolution of the two components that combined generate the observed kinetic trace shape. The dashed trace is the modulus of the negative component to help visualise the detected signal.*"

(3) I suggest that the authors should give more pump fluence dependent measurements and discussion in the paper.

We thank the Reviewer for this comment. Unfortunately, we couldn't carry out fluence-dependent measurements over significant laser fluencies because the films were unstable. We saw clear damage signs at higher fluencies that couldn't be avoided by moving the laser spot. Moreover, the films were not homogeneous, which restricted us from where we could measure in the sample. This is expected due to the fast crystalization of the film, as observed by several reports, including Wang et al. J. Mater. Chem. A 2018, 6, 21435.

(4) Please provide more information and discussion on the different temperature dependence of the positive and negative components in Fig. 6 and Fig.7.

We thank the Reviewer for this comment. The temperature dependence measurements were performed exclusively to help us discriminate between models. Detailed temperature dependence measurements were out of the manuscript's scope. However, we want to reiterate that CuI undergoes a phase transition at relatively low temperatures, precluding significant

studies with temperatures as a variable. We expanded a bit on the comments related to figures 6 and 7 throughout the text, which are highlighted in blue,

jz-2022-037206.R2

Name: Peer Review Information for "Carrier Dynamics in Solution-Processed CuI as a P-Type Semiconductor: the Origin of Negative Photoconductivity"

Second Round of Reviewer Comments

Reviewer: 2

Comments to the Author

Authors undoubtedly improved the abstract and the discussion in the revised manuscript. They also replied almost to all comments of the reviewers. If there was no possibility to measure the pump fluence dependence of the samples that is ok, as long as it is acknowledged as such in the manuscript. In other words, the authors should explicitly say that what is the pump fluence in current measurement. However, there was no further measurements to ensure this in the unstable sample.

Author's Response to Peer Review Comments:

Answers to Reviewer's Comment:

Reviewer: 2

Authors undoubtedly improved the abstract and the discussion in the revised manuscript. They also replied almost to all comments of the reviewers. If there was no possibility to measure the pump fluence dependence of the samples that is ok, as long as it is acknowledged as such in the manuscript. In other words, the authors should explicitly say that what is the pump fluence in current measurement. However, there was no further measurements to ensure this in the unstable sample.

We thank the Reviewer for the recognition of the submitted work's worthiness and novelty,as well as the endorsement for publication. We would also like to thank the Reviewer's time dedicated to the revision and helpful comment.

We added the laser fluence used for that specific measurement to each figure in the manuscript.

We also added to the supporting information the following:

Meaningful fluence-dependent measurements were not feasible because the films were unstable. There were clear damage signs at higher fluencies that could not be avoided by moving the laser spot. Moreover, the films were not homogeneous, restricting where one could perform the measurements in the film. This is expected due to the fast crystalization of the CuI film, as observed by several reports, including Wang et al. J. Mater. Chem. A 2018, 6, 21435.'
